# Physical activity among children: objective measurements using Fitbit One^®^ and ActiGraph

**DOI:** 10.1186/s13104-017-2476-1

**Published:** 2017-04-20

**Authors:** Lotta Hamari, Tiina Kullberg, Jukka Ruohonen, Olli J. Heinonen, Natalia Díaz-Rodríguez, Johan Lilius, Anni Pakarinen, Annukka Myllymäki, Ville Leppänen, Sanna Salanterä

**Affiliations:** 1 Department of Nursing Science, University of Turku, 20014 Turku, Finland; 2 Department of Information Technology, University of Turku, 20014 Turku, Finland; 3 Paavo Nurmi Centre & Department of Physical Activity and Health, University of Turku, Kiinamyllynkatu 10, 20520 Turku, Finland; 4 Turku Centre for Computer Science (TUCS), Department of Information Technologies, Åbo Akademi University, Joukahaisenkatu 3-5 A, 20520 Turku, Finland; 5 Health and Well-being Unit, Turku University of Applied Sciences, Ruiskatu 8, 20720 Turku, Finland; 6 Turku University Hospital, Kiinamyllynkatu 4–8, 20521 Turku, Finland

**Keywords:** Accelerometer, Physical activity, Motor activity, Movement, Children, Investigative techniques

## Abstract

**Background:**

Self-quantification of health parameters is becoming more popular; thus, the validity of the devices requires assessments. The aim of this study was to evaluate the validity of Fitbit One step counts (Fitbit Inc., San Francisco, CA, USA) against Actigraph wActisleep-BT step counts (ActiGraph, LLC, Pensacola, FL, USA) for measuring habitual physical activity among children.

**Design:**

The study was implemented as a cross-sectional experimental design in which participants carried two waist-worn activity monitors for five consecutive days.

**Methods:**

The participants were chosen with a purposive sampling from three fourth grade classes (9–10 year olds) in two comprehensive schools. Altogether, there were 34 participants in the study. From these, eight participants were excluded from the analysis due to erroneous data. Primary outcome measures for step counts were Fitbit One and Actigraph wActisleep-BT. The supporting outcome measures were based on activity diaries and initial information sheets. Classical Bland–Altman plots were used for reporting the results.

**Results:**

The average per-participant daily difference between the step counts from the two devices was 1937. The range was [116, 5052]. Fitbit One gave higher step counts for all but the least active participant. According to a Bland–Altman plot, the hourly step counts had a relative large mean bias across participants (161 step counts). The differences were partially explained by activity intensity: higher intensity denoted higher differences, and light intensity denoted lower differences.

**Conclusions:**

Fitbit One step counts are comparable to Actigraph step counts in a sample of 9–10-year-old children engaged in habitual physical activity in sedentary and light physical activity intensities. However, in moderate-to-vigorous physical activity, Fitbit One gives higher step counts when compared to Actigraph.

## Background

Approximately 80% of children do not engage in the recommended level of physical activity (PA) [1, 2]. Instruments for measuring PA accurately are needed by both clinical and research communities. In addition, self-quantification of health parameters in everyday life and health care is becoming more popular, which further increases the demand for accurate devices.

Accelerometry is the most commonly used objective PA measure for children and adults [3, 4]. While there are several accelerometer manufacturers and brands, ActiGraph’s (ActiGraph, LLC, Pensacola, FL, USA) products are currently the most widely used and validated devices in studies exploring children’s PA [4–6]. Fitbit One, in turn, (Fitbit Inc., San Francisco, CA, USA) is a relatively new device that has been developed for consumer use.

Yet, the devices used for research purposes are usually more expensive than devices designed for consumer use. In April 2016, a single Fitbit One tracker cost approximately US $100, including the associated holder clip, wrist band, charging cable, and auxiliary synchronization device. In addition, the Fitbit application programming interface is provided free of charge [7]. Actigraph wActisleep-BT, in contrast, cost around US $225 and the associated software requires an additional investment of US $1695 [5]. Moreover, at least one software solution is required to process, manage, and analyze Actigraph-data. Thus, when there still is a relatively high price difference between the research-level and consumer-grade devices, it is worth to evaluate the validity of lower-cost variants.

The features, including validity of Fitbit One activity trackers have been investigated in six prior studies [8–13]. However, none of these studies included children [14], and only one study was carried out in free-living conditions [10]. In general, the existing evidence on the validity of Fitbit One devices remains inconclusive and even contradictory. Step counts for adults have been observed as valid, although Fitbit One devices tend to be inaccurate for measuring distance [13] and moderate-to-vigorous physical activity [10]. When placed at the ankle, Fitbit One has been observed to provide valid step counts of older adults at slow speeds [11]. Storm et al. and Diaz et al. found that Fitbit One underestimated step counts during treadmill walking and running [9, 12]. In contrast, ActiGraph devices have been extensively validated and used in PA research among children [4–6].

As there is insufficient evidence of the use of Fitbit One in measuring PA among children, the aim of this study was to evaluate the validity of Fitbit One step counts against Actigraph wActisleep-BT step counts for measuring habitual physical activity among children.

## Methods

### Study design

The study was implemented as a cross-sectional experimental design for comparing the consumer-grade Fitbit One accelerometer to the research-level accelerometer Actigraph in free-living conditions in children.

### Participants and recruiting process

Participants were chosen with a purposive sampling method from three fourth grade classes in two comprehensive schools located in Turku, Finland. The eligibility criteria were: (a) 9–10-years of age and (b) no chronic diseases. The existing research [9, 10, 13, 15–18], showed us direction when deciding the sample size, however the lack of priori power calculation is acknowledged as a limitation. The sample size was limited to thirty participants. Altogether, 34 participants took part in the study. The data were collected during March–May 2015.

The researcher and a contact teacher started the recruitment process by distributing written research information and an initial information sheet to the children. The children were asked to give the material to their parents for discussion. The material included phone numbers and other contact information of the research team. Families were encouraged to contact the researcher for any queries. Those who were willing to participate in the study brought the initial information sheet and the signed consent back to the teacher who forwarded these to the researcher. After these preliminaries, the researcher met the children during a school day, handling the individually programmed accelerometer devices to the children. The researcher also educated the children about the study procedures.

### Instruments

#### Initial information sheet and the activity diary

The initial information sheet contained questions about demographic data (namely, age, weight, and height) and was filled in by parents. This data was used to program the devices for each child individually prior to the study time during which the participants also filled an activity diary together with their parents. In particular, the children recorded the times they had the accelerometers on and off. This procedure ensured accurate timings, more efficient data handling, and evaluation of the discrepancies in the processed data [19]. If a child did not record the wearing time in his or her diary, the corresponding activity times were defined according to the times Actigraph had registered movement.

#### Fitbit One and Actigraph devices

Fitbit One activity monitor is a triaxial accelerometer that estimates step count, distance traveled, calories burned, stairs climbed, active time, and sleep time. The weight of the device is 8 g. The data from the tracker is wirelessly uploaded to the software via Bluetooth [7].

ActiGraph wActisleep-BT provides data on PA and sleep/wake condition. ActiGraph wActisleep-BT estimates raw acceleration, steps taken, energy expenditure, PA intensity, metabolic equivalent rates, subject position, total sleep time, sleep efficiency, and ambient light levels. The weight of the device is 19 g. To see and analyze the data from wActisleep-BT, a so-called ActiLife software is needed. Bluetooth is used for synchronization [20]. Based on the manufacturer’s information, the ActiGraph wActisleep-BT device in research use is identical with a better-known model, wGT3X-BT. However, wGT3X-BT does not include the sleep functions.

### Measurements

The participants carried the two waist-worn accelerometers (Fitbit One and Actigraph) during five consecutive days, from Wednesday to Sunday. This time period was chosen based on a recommendation that at least one day from a weekend should be included for gathering accelerometer data recorded by school children. [21, 22]. Regarding the length of data gathering periods, a minimum of four days is considered acceptable [23], and even three days may be satisfactory in case of young children [6, 24]. Both activity monitors were set to collect data in one min epochs. The choice was necessary as Fitbit One does not allow changing the predefined recording epoch.

### Data analysis

The collected dataset had to be processed by excluding observations from participants whose devices were found to be faulty during the study time. The defects were related to the Fitbit One device clocks, which stopped randomly for a few minutes or even for a few hours. These defects prevented pairing with the corresponding Actigraph devices. Two defective Fitbit One products were identified. These were worn by seven participants. Furthermore, one participant had to be excluded because the associated devices did not record any activity during the third day. All in all, the devices and recordings from 26 participants were qualified for the data analysis.

For comparing the recordings, the step counts were scaled to hourly averages. This scaling was necessary because the individual sample sizes varied from a participant to another due to the measurement periods reported in the activity diaries. Given this scaling, the classical Bland–Altman plot [25] was used to assess the mean bias and the limits of agreement.

## Results

The mean age of participants (n = 26, 15 boys, 11 girls) was 9.6 years. The participants’ mean height was 140 cm (with a range 132–150 cm) and mean body weight was 34 kg (range 26–50 kg). The mean body mass index for children was 22.1 (range 17.8–29.5).

The wear-time (per-subject sample sizes) varied due to the different time periods reported by the children in their activity diaries. On average, 3581 min were recorded by each child. The range varied from 2889 to 3925 min.

**Fig. 1:**
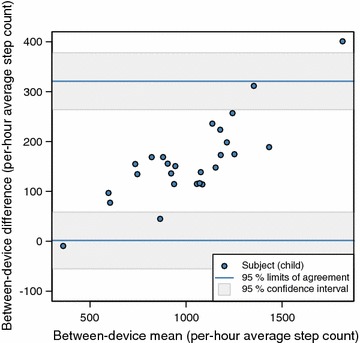
Bland–Altman plot (mean step counts/hour). Bland–Altman plot shows large mean individual biases and 95% limits of agreement between Fitbit One and Actigraph. The mean bias across children was 161.2 step counts per hour. Fitbit One gave higher step counts for all but the least active participant

Bland–Altman plot (Fig. 1) showed large mean individual biases and 95% limits of agreement between the two devices. The mean bias across children was 161.2 step counts per hour, while the limits of agreement ranged from 1.6 to 320.7 step counts per hour. Fitbit One gave higher step counts for all but the least active participant. While the step counts are not normally distributed at an individual level, it is worth noting that both the hourly differences and the hourly means are approximately normal according to the Shapiro–Wilk normality test (the *p* values for testing the null hypotheses of normality are 0.098 and 0.790, respectively).

There was a linear trend between the differences and the means; increasing mean physical activity (step counts) increased the measurement difference between the two devices. For examining this trend further, the observations were classified into sedentary (0–100 counts per minute), light (101–2295 counts per minute), moderate (2296–4011 counts per minute), and vigorous (over 4 012 counts per minute) activity. These activity intensity classes were chosen since they have performed well for classifying physical activity intensity of young children [26]. The fourfold grouping further illustrated that the disagreement increases as the activity intensity increases.

**Fig. 2:**
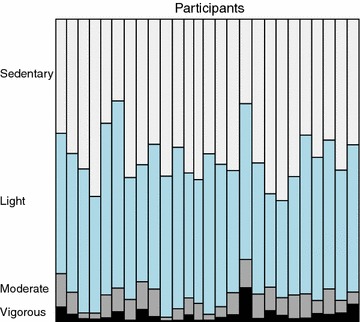
Relative share of minutes spent in activity classes (%). *Figure* shows the relative amount of minutes spent in the four activity classes for each participant. The children spent most of their time in the sedentary and light activity classes

The children spent most of their time in the sedentary and light activity classes. This observation can be seen from Fig. 2, which shows the relative amount of minutes spent in the four activity classes for each participant.

**Fig. 3:**
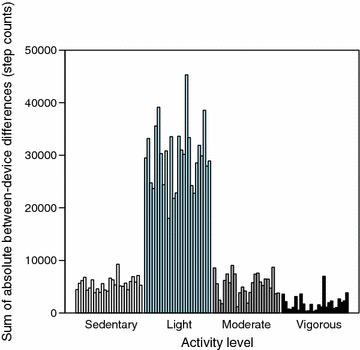
Absolute difference across activity classes. The absolute per-subject sums of the differences between the devices are largest in the light activity class

**Fig. 4:**
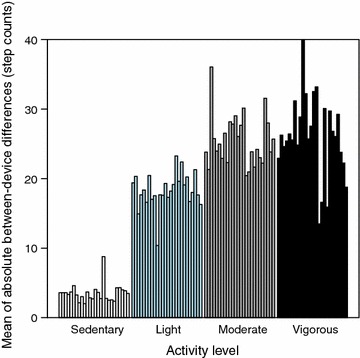
Average difference across activity classes. Disagreement between Fitbit One and Actigraph step counts increases steadily across the activity classes. The *y-axis* represents the absolute per-subject mean differences between the devices (scaled by the minutes spent in each class)

Since the data contains a large amount of light activity, the absolute per-subject sums of the differences between the devices are also largest in the light activity class (see Fig. 3). When these sums are scaled by the minutes spent in each class, however, the disagreement increases steadily across the classes (see Fig. 4 within which the *y*-axis represents the absolute per-subject mean differences between the devices). To summarize, a portion of the observed differences is explained by the increasing disagreement between the two devices when the physical activity intensity is increased.

Lastly, it must be mentioned that all Actigraph devices were technically functioning, but two defective Fitbit One products were identified during the study.

## Discussion

The aim of the study was to compare Fitbit One step counts against Actigraph wActisleep-BT step counts for measuring habitual physical activity of healthy 9–10-year-old children. In the present study, we found that the hourly step counts showed a mean bias of 161 step counts according to the Bland–Altman plot. The differences were partially explained by the activity intensity: higher intensity denoted higher differences, and light intensity denoted lower differences. Fitbit One overestimates the step counts of moderate-to-vigorous physical activity compared to Actigraph. Interestingly, this result is in discrepancy with previous results with adult samples where Fitbit One underestimated step counts compared to manual counting of steps in laboratory circumstances in walking and running [9]. More essential is that when measured in free-living conditions, our results are in accordance with previous findings [14]. In particular, Ferguson et al. (2015) suggest that Fitbit One overestimates the step counts compared to Actigraph GT3X+ in a setting involving healthy adults in free-living conditions [10]. It must be noted that these studies were conducted with adult samples [9, 10].

The advantage of our results is that they provide a preliminary framework for putting the Fitbit One step counts for relation with Actigraph step counts in measuring children’s physical activity in free-living conditions. However, with the methodology sketched, it is impossible to state conclusions regarding sensitivity and specificity [27]. In other words, how far the movement Fitbit or Actigraph detects must be “true” movement.

A few limitations of the study must be mentioned. Firstly, a missing “golden standard” measure of energy expenditure is recognized as a limitation. This said, our aim was to evaluate Fitbit in free-living conditions during 5 days, and thus, we begun the work by comparing Fitbit One with a research-grade accelerometer. Secondly, two defective Fitbit One products (worn by seven participants) were identified, leading to data loss. The defects were related to the Fitbit One device clocks. Other studies measuring the validity of consumer-level activity trackers have also reported analogous data issues [14]. Losing valuable data due to defective devices is a matter of feasibility, and needs to be addressed when making decisions on instruments and sample sizes for research use. Thirdly, the sample size of this study was estimated based on previous literature. In particular, power analysis was not conducted prior to data collection. Fourthly, the body weight and height of the children were obtained from parents and not measured. This might give rise to some inaccuracy when configuring the settings in the devices for the actual body weight and height.

The study has also strengths. This is the first study to report on the validity of Fitbit One for measuring children’s habitual physical activity. Also, the data collection took place in free-living conditions in order to gain knowledge about accelerometers’ accuracy in real-world conditions, which has been recommended for validation studies [14, 26]. All in all, further research is required for validating Fitbit One for measuring the physical activity of children.

## Conclusions

Fitbit One step counts are comparable to Actigraph step counts in 9–10-year-old children in habitual physical activity in low intensities. However, for activities of high intensity, Fitbit One overestimates the step counts compared to Actigraph.
